# Negative Role of the Environmental Endocrine Disruptors in the Human Neurodevelopment

**DOI:** 10.3389/fneur.2016.00143

**Published:** 2016-08-30

**Authors:** Luca Roncati, Veronica Termopoli, Teresa Pusiol

**Affiliations:** ^1^Provincial Health Care Services, Institute of Pathology, Santa Maria del Carmine Hospital, Rovereto, Trentino, Italy; ^2^Department of Diagnostic and Clinical Medicine and Public Health, University of Modena and Reggio Emilia, Modena, Modena, Italy; ^3^LC-MS Laboratory, Department of Earth Sciences, Life and Environment (DiSTeVA), University of Urbino, Urbino, Italy

**Keywords:** endocrine disruptors, gas chromatography–mass spectrometry, stillbirth, sudden intrauterine unexplained death syndrome, sudden infant death syndrome, pesticides, neurodevelopment

## Abstract

The endocrine disruptors (EDs) are able to influence the endocrine system, mimicking or antagonizing hormonal molecules. They are bio-persistent for their degradation resistance in the environment. Our research group has investigated by gas chromatography–mass spectrometry (GC–MS) the EDs presence in 35 brain samples, coming from 27 cases of sudden intrauterine unexplained death syndrome (SIUDS) and 8 cases of sudden infant death syndrome (SIDS), collected by centralization in the last year (2015). More in detail, a mixture of 25 EDs has been subjected to analytical procedure, following standard protocols. Among the target analytes, some organochlorine pesticides, that is α-chlordane, γ-chlordane, heptachlor, *p*,*p*-DDE, *p*,*p*-DDT, and the two most commonly used organophosphorus pesticides (OPPs), chlorpyrifos and chlorfenvinfos, have been found in seven and three samples, respectively. The analytical procedure used to detect the presence of environmental EDs in cortex samples has been successfully implemented on SIUDS and SIDS victims. The environmental EDs have been found to be able to overcome the placental barrier, reaching also the basal ganglia assigned to the control of the vital functions. This finding, related to the OPPs bio-persistence, implies a conceptual redefinition of the fetal–placental and fetal blood–brain barriers: not real safety barriers but simply time-deferral mechanisms of absorption.

## Introduction

According to the definition of the *United Nations Environment Programme* and of the *World Health Organization*, the endocrine disruptors (EDs) are exogenous substances that alter the function(s) of the endocrine system and, consequently, cause adverse effects in an intact organism (1). They can be found in pesticides, metals, additives or contaminants of food, deep and superficial waters, and personal care products (1). A growing role of EDs has been ascertained in many diseases and particular attention has been recently focused on maternal, fetal, and childhood exposure (2). In fact, EDs have the capacity to interfere with the tissue and organ development and the related functions (3, 4). For example, the exposure to EDs has been associated with female reproductive dysfunctions (endometriosis, polycystic ovary syndrome, and infertility) and breast cancer risk or progression (5–9). The members of the *Endocrine Society* have established that exist scientific evidences for the association of EDs exposure to the following conditions: (I) obesity and diabetes; (II) dysfunction of female reproduction; (III) dysfunction of male reproduction; (IV) hormone-sensitive female cancers; (V) prostate diseases; (VI) thyroid dysfunctions; and (VII) diseases of neurodevelopment and neuroendocrine systems (10). Our attention has been focused to search environmental EDs in brain samples of sudden intrauterine death syndrome (SIUDS) and sudden infant death syndrome (SIDS) victims, coming from agriculture areas of the Northeast Italy, in whom a complete autopsy and a detailed analysis of the clinical history have ruled out any other rare and possible cause of death (11–15).

## Materials and Methods

We have analyzed 35 cases of sudden perinatal death, that is 27 SIUDS (age 25–41 gestational weeks) and 8 SIDS (age 2 h–6.5 months), coming from the Northeast Italy, Autonomus Province of Trento included, collected by centralization in the last year (2015). Significant samples of cerebral cortex of the victims have been sent to LC-MS Laboratory of the University of Urbino for gas chromatography–mass spectrometry (GC–MS) investigation. All the samples have been frozen at −20°C until analysis, which has been performed according to our previously published method (16). Briefly, each defrosted brain sample has been weighed (approximately 0.5 g) and homogenized with 2 mL of *n*-hexane to obtain a dense rich supernatant. The homogenized tissue has been transferred into a solid phase extraction (SPE) cartridge containing 500 mg of C18 sorbent, in order to retain most of the matrix impurities and to release the compounds of interest with hexane. The SPE cartridge has been conditioned with 4 mL of *n*-hexane, before purification step, and it has been washed with 1 mL of *n*-hexane, followed by 1 mL of dichloromethane after elution step. The extraction method has been developed and validated in terms of accuracy, precision, limit of quantification (LOQ), limit of detection (LOD), and linearity. Nine isotopically labeled internal standards (ISTDs) have been used for method validation. The validated method has been subsequently applied to human tissues from SIUDS and SIDS victims.

### Chemicals and Materials

A mixture of 20 organochlorine compounds (EPA CLP mix), chlorpyrifos, chlorfenvinfos, captan, boscalid, and bisphenol A have been purchased from Sigma-Aldrich (Milan, Italy). All solvents used (*n*-hexane and dichloromethane) have been supplied by Merck (Suprasolv 99% purity, Merck, Darmstadt, Germany). Stock solutions have been prepared in *n*-hexane at 100 μg/mL concentration. A standard mixture containing all compounds (25 specific EDs and 9 ISTDs) has been prepared by appropriate dilution and stored at 4°C in the dark. SPE cartridges HyperSep-C18 (500 mg/6 mL) have been purchased from Thermo Scientific (Bellefonte, PA, USA).

### Instrumentations

The analyses on the extracted samples have been performed by an Agilent Technologies gas chromatograph 6890N, equipped with a single quadrupole mass spectrometer 5975C TAD/MS, working in electron ionization. All brain tissues have been subjected to analytical procedure in order to determine the level of the 25 selected compounds. The chromatographic separation has been carried out using an HP-5MS (Agilent J&W GC columns, Folsom, CA, USA), i.d. 30.0 m × 0.25 mm, containing 5% phenyl-methylsiloxane, with a phase thickness of 0.25 μm. As carrier gas, helium at 1 mL/min (constant flow) has been adopted. The GC oven temperatures have been programmed as follows: 80°C held for 1 min, ramped at 30°C/min to 180°C, ramped at 3°C/min to 225°C, held for 4 min, ramped at 20°C/min to 300°C, and held for 4.08 min (total acquisition time: 25 min). Splitless sample injection of 1 μL at 250°C has been selected. The transfer line and ion source temperature have been kept at 290 and 300°C, respectively.

## Results

Among the target analytes, organochlorine pesticides (OCPs) and organophosphorus pesticides (OPPs) have been detected in part-per-billion (ppb) in 7 and 3 out of 35 cortex samples coming from SIUDS (7 cases) and SIDS (3 cases) victims, respectively. The following OCPs and OPPs have been found: α-chlordane, γ-chlordane, heptachlor, *p*,*p*′-dichlorodiphenyldichloroethylene (DDE), *p*,*p*′-dichlorodiphenyltrichloroethane (DDT), chlorfenvinfos, and chlorpyrifos. More in detail, heptachlor, DDE, and DDT have been detected in three separate cases, while chlorfenvinfos and chlorpyrifos in association with α-chlordane or γ-chlordane. Today, chlorfenvinfos and chlorpyrifos are the two most commonly used organophosphate pesticides for pest control in intensive agricultural areas, such as the Northeast Italy is. These non-persistent compounds, also called contemporary-use pesticides, are currently available in place of organochlorine compounds, banned since 1980s. However, the detection of DDE and DDT confirms their degradation resistance over the years, and their extensive use has exposed the population to insidious environmental administrations. Fetuses, newborns, and pregnant women appear to be the most vulnerable subjects to these exposures.

## Discussion

The detection of some EDs in 10 positive samples out of 35 fetal and neonatal brain tissues confirms the possibility of these chemicals to pass from mother to fetus, overcoming the fetal–placental and fetal blood–brain barriers, not real safety barriers but simply time-deferral mechanisms of absorption, and to be collected in brain tissue (2–4). Here, they can give origin to impairment of receptorial expression, such as orexin, and to development alterations, especially of the basal ganglia, the major controllers of basic vital functions (17, 18). This is consistent with further literature data, which report the presence of these selected pollutants in other human tissues (19, 20). Moreover, by our preliminary data (not shown), they seem to be able to interfere with circulating mitochondrial DNA (mtDNA), causing mitochondrial dysfunction in SIUDS and SIDS. It is already known that during normal pregnancy mtDNA level significantly decreases in different trimesters (21). In case of intrauterine growth restricted pregnancy, placental and blood mtDNA content has been found significantly increased, if compared to normal pregnancies, probably for a compensatory action (22). On the other hand, circulating mtDNA content has been significantly found decreased in gestational diabetes mellitus (23) and in HELLP syndrome (24), indicating a reduction of the mitochondrial activity. For all these reasons, environmental EDs should be systematically searched during autopsy in order to establish a possible correlation with SIUDS and SIDS events. Moreover, it is interesting to remark that seven brain samples have shown the presence in ppb levels of several OCPs and three samples have disclosed the presence of two OPPs, chlorpyrifos and chlorfenvinfos, the two most common pesticides used in apples cultivation. These findings are in accordance with the environmental diffusion of contaminants in intensive agricultural areas represented by the Northeast Italy (25). The *Italian National Institute for Environmental Protection and Research* (ISPRA) has published a detailed report on the presence of pesticides in surface and ground water in the period 2013–2014 (26). The aim of this national investigation has been to provide reliable information on the water quality in relation to these substances. The report has been the outcome of a complex activity, also involving the *Regional Agencies for Environmental Protection* (ARPA) and the *Provincial Agencies for Environmental Protection* (APPA). The data collected by these institutions have concerned with recovery, frequency, and distribution of pesticides on the whole Italian territory. Moreover, the measured concentrations have been compared with the legal threshold fixed by the European and National legislation: the Standard of Environmental Quality for the surface water (dir. acque 2008/105/CE; D.Lgs 152/2006). In the Northeast Italy, the Autonomous Province of Trento has provided 70 monitoring stations: 764 samples have been collected and 64,283 measurements have been performed. Thanks to this extensive analysis, 102 substances have been researched, and 33 of them have been detected in 18–23% samples of surface waters. Among these, boscalid, dimetomorf, fluopicolide, and chlorpyrifos have been resulted the chemicals most frequently found. Unsurprisingly, chlorpyrifos is one of the EDs detected also in our examined brain samples. In conclusion, for the first time in literature, our research group has successfully demonstrated the presence of pesticides at ppb levels in cortex samples of SIUDS and SIDS victims; it is not ethically possible to compare the obtained results with brain samples coming from healthy subjects, and, consequently, it will not be possible to draw up threshold values for the future. However, even though the contaminants have been detected at ppb levels, their presence can be considered significant due to their certified high degree of toxicity and to fetal/infant known vulnerability.

## Author Contributions

LR: study design and supervision; VT: data acquisition; and TP: drafting of the manuscript.

## Conflict of Interest Statement

The authors declare that the research was conducted in the absence of any commercial or financial relationships that could be construed as a potential conflict of interest.
